# A prospective observational cohort study of covid-19 epidemiology and vaccine seroconversion in South Western Sydney, Australia, during the 2021–2022 pandemic period.

**DOI:** 10.1186/s12882-024-03560-8

**Published:** 2024-04-12

**Authors:** Daniela Potter, Jason Diep, Colleen Munro, Noelle Lin, Ramon Xu, Jeffrey Wong, Robert Porritt, Michael Maley, Hong Foo, Angela Makris

**Affiliations:** 1 University of Western Sydney, Sydney, NSW Australia; 2 University of New South Wales, Sydney, NSW Australia; 3 Department of Renal Medicine, Liverpool Hospital, Liverpool, Sydney, NSW Australia; 4 Department of Microbiology and Infectious Diseases, NSW Health Pathology, Liverpool, NSW Australia

**Keywords:** Kidney Transplantation, COVID-19, COVID-19 Epidemiology, COVID-19 Vaccination, COVID-19 Vaccination Seroconversion

## Abstract

**Background:**

It is known that COVID-19 disproportionally adversely affects the immunocompromised, including kidney transplant recipients (KTR), as compared to the general population. Risk factors for adverse outcomes and vaccine seroconversion patterns are not fully understood. Australia was uniquely positioned to reduce initial case numbers during the 2021–2022 pandemic period due to its relative isolation and several significant public health interventions. South-Western Sydney Local Heath District was one of the predominant regions affected.

**Methods:**

A single centre, prospective cohort study of prevalent renal transplant recipients was conducted between 25th July 2021 and 1st May 2022. Baseline characteristics, COVID-19 vaccination status, COVID-19 diagnosis and outcomes were determined from the electronic medical record, Australian vaccination register and Australian and New Zealand Dialysis and Transplant Registry. Assessment of vaccine-induced seroconversion was assessed with ELISA in a subpopulation. Analysis was performed using SPSS v.28.

**Results:**

We identified 444 prevalent transplant recipients (60% male, 50% diabetic, median age 58 years (Interquartile range (IQR)21.0) and eGFR 56 ml/min/1.73m^2^ (IQR 21.9). COVID-19 was identified in 32% (*n* = 142) of patients, of which 38% (*n* = 54) required hospitalisation and 7% (*n* = 10) died. At least one COVID-19 vaccination was received by 95% (*n* = 423) with 17 (4%) patients remaining unvaccinated throughout the study period. Seroconversion after 2 and 3 doses of vaccine was 22% and 48% respectively. Increased COVID-19 related deaths were associated with older age (aOR 1.1, 95% CI 1.004–1.192, *p* = 0.040), smoking exposure (aOR 8.2, 05% CI 1.020-65.649, *p* = 0.048) and respiratory disease (aOR 14.2, 95%CI:1.825–110.930, *p* = 0.011) on multi-variable regression analysis. Receipt of three doses of vaccination was protective against acquiring COVID-19 (aOR 0.48, 95% CI 0.287–0.796, *p* = 0.005) and death (aOR 0.6, 95% CI: 0.007–0.523, *p* = 0.011), but not against hospitalisation (*p* = 0.32). Seroconversion was protective for acquiring COVID-19 on multi-variable regression independent of vaccination dose (aOR 0.1, 95%CI: 0.0025–0.523, *p* = 0.011).

**Conclusions:**

COVID-19 was associated with a high mortality rate. Older age, respiratory disease and prior smoking exposure may be risk factors for increased mortality. Vaccination of 3 doses is protective against acquiring COVID-19 and death, however not hospitalisation. Antibody response is protective for acquiring COVID-19, however seroconversion rates are low.

**Supplementary Information:**

The online version contains supplementary material available at 10.1186/s12882-024-03560-8.

## Introduction

It is known that COVID-19 disproportionally adversely affects the immunocompromised, including kidney transplant recipients (KTR), as compared to the general population. The advent of specific COVID-19 therapies and novel vaccination improved outcomes, however mortality rates for organ transplant recipients from large cohort studies remained as high as 14% into 2021 [1, 2]. Factors predicting mortality are not fully understood but age, cardiovascular disease, diabetes, and certain immunosuppression regimens have been suggested [1, 3–7]. KTRs were prioritised for vaccine administration, however, were not included in original vaccination trials [8, 9]. Subsequent data suggests conventional 2-dose regimens are insufficient for KTRs, with 3 doses potentially ineffective against later strains such as BA.1 (Omicron) [10–12]. The primary course of vaccination was extended, between March 2021 and July 2022, to 5 doses in Australia, however adequate ongoing vaccination strategies are unclear [13].

## COVID-19 in Australia and South Western Sydney

Australia was protected from high case numbers during the early phases of the pandemic due to its geographical isolation, strict initial international border controls and aggressive case tracking. Although Australia comprises 6 states and two territories, each have a significant degree of independence and power in health policy making. Those states with low case numbers throughout 2020–2022, such as South and Western Australia, maintained strict international and interstate border controls, but relaxed internal restrictions with almost near normal, pre-COVID, living conditions. They enacted limited, “snap lockdowns” in response to small numbers of detected cases to keep COVID-19 suppressed, until the majority of the population could be vaccinated [14]. Within New South Wales, however, several significant outbreaks occurred in 2021–2022, prompting repeated modification of public orders and prolonged periods of community lockdown and restrictions [15]. South Western Sydney Local Health District (SWSLHD) was one of the first areas in New South Wales (NSW) to be affected by COVID-19, and experienced one of the higher reported case numbers and the highest reported deaths of any Local Health District in NSW [16]. In response to the high rates of infection, SWSLHD experienced the most restrictive lockdown regulations in NSW during the pandemic period. During the second NSW wave in 2021, several local government areas within SWSLHD were classed as “areas of concern” and had additional public orders imposed, including: a stay at home order, restrictions on entering or leaving a district except for specific work exemptions (which required a permit), not allowed to travel more than 5 km for exercise, mandatory mask wearing outside and, at one point, a 9pm to 5am curfew [17]. August and September 2021 was associated with peak B.1.617.2 (Delta) wave incidence, followed by peak BA.1 (Omicron) in January 2022 [16]. COVID-19 vaccination was available for immunosuppressed individuals in Australia from 22nd March 2021 [18]. There is also a large burden of chronic kidney disease (CKD), with SWSLHD accounting for approximately 3.3% of prevalent KTRs in Australia. SWSLHD is also diverse, multiethnic population with 54% of people speaking a language other than English, predominantly Arabic or Vietnamese, and 43% of the population were born overseas, in comparison to 29% to the rest of NSW [19, 20].

This study was conducted in the 2nd to 3rd year of the pandemic, during two dominant strain outbreaks, B.1.617.2 (Delta) and BA.1.(Omicron), after vaccination was available for all recipients [15]. Our objective was to ascertain the impact of COVID-19 on KTRS, with a focus on acquisition, hospitalisation, and mortality from COVID-19, and to perform a serology assessment of vaccine seroconversion.

## Methods

### Study design

A single centre (SWSLHD) prospective cohort study of prevalent kidney transplant recipients was undertaken between 25th July 2021 and 1st May 2022.

### Setting

The study was commenced prospectively, coinciding with the onset of rising COVID-19 transmission and initiation of community stay at home orders. After restrictions had ended, vaccination numbers had increased, and it was clear no further public health orders were likely to be initiated, the study was terminated. All KTRs were strongly encouraged to receive vaccination throughout the study period, via national public health messaging, family practitioner support, and nephrologist advice. The Renal department at SWSLHD undertook a program at this time to encourage immunisation by developing a multi-lingual (Arabic, Vietnamese) information letter in view of the multi-ethnic population (distributed, mailed or emailed) to all KTRs and dialysis patients. A dedicated contact nephrologist was available to answer vaccination specific queries to facilitate timely immunisation.

### Participants

All prevalent KTRs, aged ≥ 18, were included in the initial observational component of the study (see ethics below). Patients were identified from an existing clinical database and cross-referenced by searching the entire health district electronic coding system for renal transplantation to reduce the risk of selection bias. After the final data collection point on 1st May 2022 the cohorts of COVID-19 positive and COVID-19 negative patients were identified.

### Variables and data sources

Baseline clinical and transplant characteristics, including: age, sex, body mass index, place of birth, smoking status, primary renal disease, co-morbidities, baseline eGFR, use of any blood thinner, prior dialysis modality and modality change during study, requirement for an interpreter, number of transplants, donor type, number of mismatches, transplant vintage, baseline immunosuppression regiment, dosage and levels and administration of Anti-thymocyte globulin, were determined from the electronic medical record, the Australian and New Zealand Dialysis and Transplant Registry (ANZDATA) records, and locally available Nephrologist letters. The date and brand of each COVID-19 vaccination is recorded into the Australian Immunisation Register and electronic health record prospectively. We obtained information on every dose of COVID-19 vaccination provided to patients. We assessed the impact of increasing vaccination dose, from 1 onwards. COVID-19 diagnosis, and outcomes were determined from the electronic medical record, including date of diagnosis, administration of sotrovimab or molpurinovir, hospitalisation and level of care for COVID-19, oxygen requirement, use of dexamethasone and other adjunctive agents including baricitinib, tocilizumab, remdesivir and sarilumab, length of stay and mortality from COVID-19.

### Bias

The combination of both ANZDATA records, local electronic health record and Nephrologist letters was utilised to reduce missing data and increase accuracy of data imputation. The study period encompassed a period of mandatory reporting of all positive COVID-19 polymerase chain results and rapid antigen tests to the NSW Health Service. In SWSLHD each positive result was reviewed by a dedicated COVID-19 Community Health service and documented in the electronic health record, which we anticipated would reduce the impact of sampling bias and missing data.

### Serology assessment

All patients were invited to participate in the post COVID-19 vaccination serology conversion assessment component of the study. At study commencement all patients received a multi-lingual text message (English, Arabic, or Vietnamese) offering participation in COVID-19 serologic conversion testing. Additional written informed consent for this component of the study was obtained from those willing to participate. Blood tests we requested to be performed at least 14 days after their 2nd and 3rd vaccine dose. These patients were planned to be analysed as a subgroup from the main cohort.

All patient serum underwent testing at NSW Health Pathology– Liverpool, using both the Roche Elecsys Anti-SARS-CoV-2 assay (“Elecsys”) and the EUROIMMUN Anti-SARS-CoV-2 QuantiVAC ELISA (“QuantiVAC”), which have different targets. The Elecsys assay is an electrochemiluminescence assay for the qualitative detection of antibodies to SARS-CoV-2 nucleocapsid protein in human serum, and is considered reflective of wild-type infection [21]. A result (cutoff index; signal to cutoff ratio) of ≥ 1.0 is considered reactive. The QuantiVAC ELISA is an enzyme immunoassay, providing quantitative in vitro determination of antibodies to the immunoglobulin class IgG against the S1 antigen and receptor binding domain of SARS-CoV-2 [22]. Detection of the anti-S1 (spike) antibody is considered to indicate either a wild-type infection or a response to vaccination. A result of < 8RU/ml was considered negative, ≥ 8-<11 RU/ml borderline and ≥ 11RU/ml positive. Utilising the results from these two assays, in conjunction with the patient’s vaccination status and any noted clinical COVID-19, it was possible to determine whether the patient’s antibody response was secondary to clinical infection or to vaccination (Supp Table 1).

**Table 1:** Baseline demographic of study cohort

Baseline Characteristics, *N* = 444*	*N* (%)
**Age**, *yrs*, *median (IQR)*	58 (21.0)
**Male Sex **(%)	267 (60)
Body Mass Index*, *kg/m*^*2*^, *median (IQR)*	26.8 (± 7.7)
**Place of Birth (%)**	
Australia/New Zealand	179 (40)
Pacific Island	33 (7)
Asia	106 (24)
North/South America	16 (4)
Middle East/Africa	57 (13)
Europe	53 (12)
**Interpreter required (%)**	119 (27)
Smoking status* (%)	
Current	13 (3)
Former	138 (31)
Never	277 (62)
**Co-morbidities %**	
Hypertension	376 (85)
Diabetes	221 (50)
Type 1	24 (5)
Type 2	197 (45)
Cardiac disease	121 (27)
Neurological disease	106 (24)
Respiratory disease	89 (20)
Haematological disease	65 (15)
Autoimmune disease	45 (10)
Liver disease	35 (8)
Current malignancy	33 (7)
Peripheral Vascular disease	22 (5)
Chronic Hepatitis B	19 (4)
Hepatitis C	5 (1)
**eGFR at study start**, *ml/min/1.73m*^*2*^, *mean (SD)*	57 (21.9)
**Prior Dialysis and renal disease**	
Prior Dialysis Modality * (%)	
Haemodialysis	174 (39)
Peritoneal Dialysis	119 (27)
Both	107 (23)
Pre-Emptive transplant	38 (9)
Modality change during study	8 (2)
**Primary Cause of Renal disease (%)**	
Glomerulonephritis	222 (50)
Diabetes	66 (15)
ADPCKD	47 (10)
Obstructive Uropathy	36 (8)
Hypertension	34 (8)
Other	39 (9)
**Transplant Information**	
Donor Type * (%)	
Deceased	308 (69)
Live	129 (29)
Related	88 (20)
Unrelated	41(9)
**Number of Transplants (%)**	
1	429 (96.6)
2	14 (3.2)
3	1 (0.2)
Mismatches * (%)	
0–1	28 (6)
2–3	94 (21)
4–6	221 (50)
**Transplant vintage**, *months, median (IQR)*	69.0 (± 111.0)
**MEDICATIONS**	
**Baseline Immunosuppression (%)**	
Prednisolone	411 (93)
Prednisolone dose, *mg, median (IQR)*	5.0 (5.0)
Mycophenolate	356 (80)
Mycophenolate dose, *mg, mean (SD)*	1193.5 (505.0)
Tacrolimus	321 (72)
Tacrolimus level, n*g/ml, median (IQR)*	6.3 (3.0)
Ciclosporin	43 (10)
Ciclosporin level, n*g/ml, median (IQR)*	134 (219)
Everolimus	42 (10)
Everolimus level, n*g/ml, mean (SD)*	5.3 (1.8)
Sirolimus	22 (5)
Sirolimus level, *ng/ml, mean (SD)*	5.8 (1.6)
Azathioprine	39 (9)
Azathioprine, *mg, mean (SD)*	83 (37.0)
**ATG**	46 (10)
**Time from ATG**, *yrs, median (IQR)*	3.0 (6.3)
**Use of Any Blood thinner**	135 (30)
COVID-19 VACCINATION DOSES*	
** 0**	17 (4)
** 1**	4 (1)
** 2**	75 (17)
** 3**	239 (54)
** 4**	105 (24)

*Missing values. BMI = 16, Smoking status = 16, Prior dialysis modality = 6, Type of transplant = 7 Mismatches = 101, Vaccination history = 4

IQR- Interquartile range, SD– Standard Deviation

To determine vaccine-induced seroconversion in patients who undertook serial testing, we reviewed the relationship of serial serum collections, vaccination and known COVID-19. The serum sample collected closest in time to the date of vaccination, with a minimum of 14 days post-vaccination was included in the analysis. If there was evidence of seroconversion from a reactive QuantiVac ELISA, subsequent reactive samples were not included. If there was evidence of seroconversion after a subsequent incrementing vaccine dose, with no known interval COVID-19, patients were considered to have vaccine-induced seroconversion at the incrementation. If there was no evidence of seroconversion, despite additional vaccine administration, the final sample was included to reflect this. Borderline results were considered as seroconverted in the context of immunocompromise.

### Ethics

This study was approved by the SWSLHD Human Research and Ethics Committee (Approval Reference: 2019/STE00860) with a waiver of consent for the initial cohort analysis of all KTRs in the district and individual informed consent for the serology component if a patient elected to participate.

### Statistical methods

Data was analysed using parametric and non-parametric tests for normally distributed and non-normally distributed variables respectively. Univariate analysis was performed with chi-squared, or Fishers test as appropriate, on categorical variables, and either independent t-test or Mann-Whitney U for continuous variables. Missing data was left as null with no imputation. Any variable with > 10% missing data was not included in any model Multi-variable binary logistic regression (backward stepwise conditional) was undertaken. Probability of entry for any variable was 0.05, removal 0.1. A goodness-of-fit test was undertaken ((Hosmer-Lemeshow test) was utilised to assess the goodness of fit and stability of the model. Statistical analysis was performed using SPSS v.28. *P* < 0.05 (2-sided) was considered significant. Strengthening the Reporting of Observational studies in Epidemiology (STROBE) guidelines were followed for the reporting of the results [26].

## Results

**Fig. 1:**
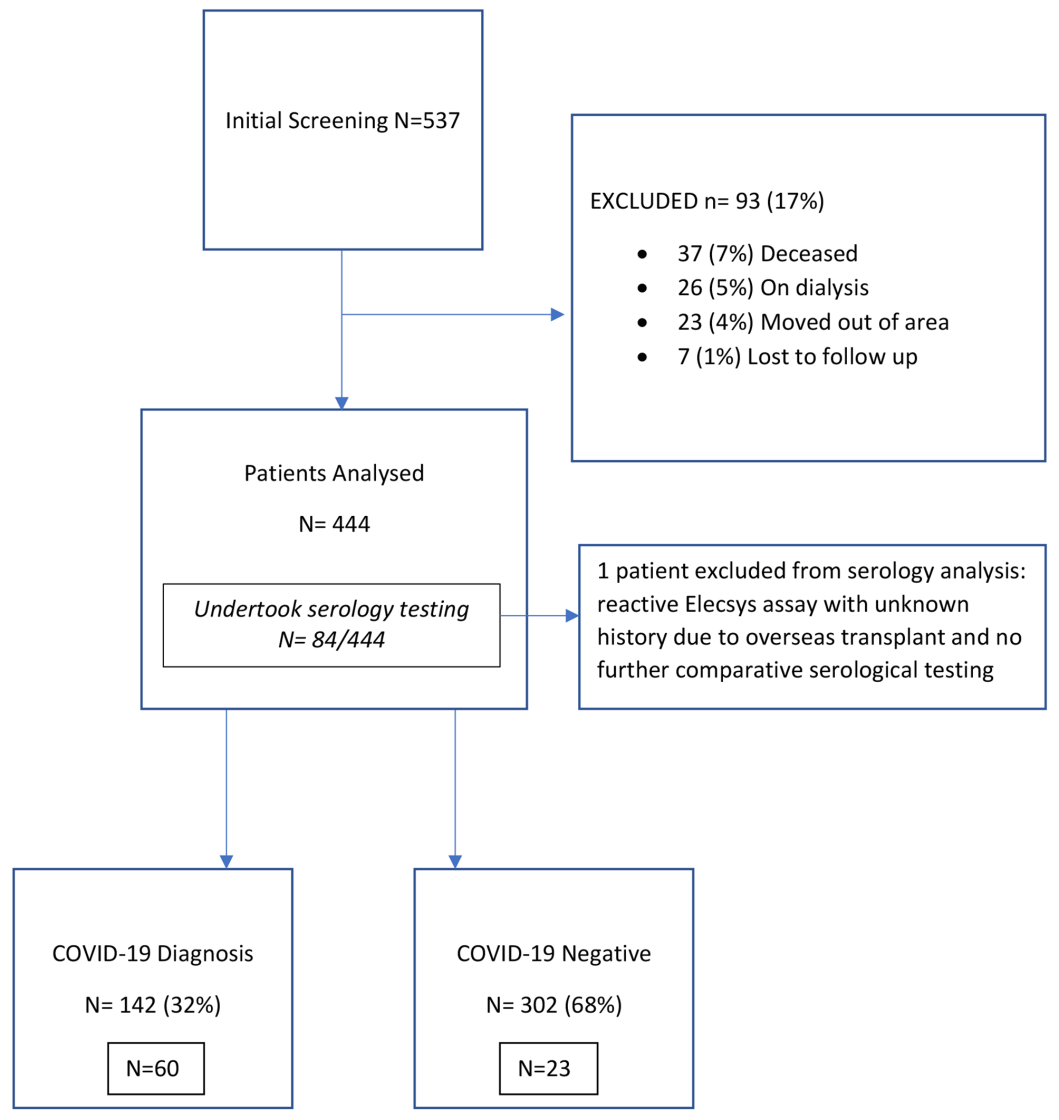
Flow chart of patient inclusion and COVID-19 diagnosis

A total of 537 patients were initially identified. After excluding patients that were deceased (*n* = 37), already on dialysis (*n* = 26), moved out of area (*n* = 23), or lost to follow up (*n* = 7), a total of 444 patients remained for analysis (Fig. 1.). A total of 84 patients elected to participate in testing for the seroconversion analysis, who were analysed as a subgroup. Baseline characteristics of the final 444 prevalent KTRs are shown in Table 1. They were predominantly male (60%), with a median age 58 years (Interquartile range [IQR]21.0) and baseline mean estimated glomerular filtration rate (eGFR) of 57 ml/min/1.73m^2^ (Standard Deviation [SD] 21.9). Patients were primarily deceased donor recipients (69%) due to glomerulonephritis (50%) or diabetes (15%), with a median transplant vintage of 69.0 months (IQR 111.0). The primary immunosuppression regimen consisted of prednisolone (93%), mycophenolate (80%), and tacrolimus (72%).

### Vaccination status

Vaccination status was acquired for 440 (99%) patients. By study end, 95% (*n* = 423) of patients had received at least 1 vaccination. The number of patients that received 1,2,3, or 4 vaccine doses was 4 (1%), 75 (17%), 239 (54%) and 105 (24%) respectively. 17 (4%) patients remained unvaccinated throughout the study period. The vaccines administered included Pfizer BioNTech BNT162b2 (70%), AstraZeneca ChAdOx1 nCoV-19 (26%) and Moderna mRNA-1273 (3%) (Supp Table 2).

**Table 2:** Univariate associations for COVID-19 diagnosis, hospitalisation and death among kidney transplant recipients

Variable of interest	No COVID-19 Detected *N* = 302	COVID − 19 Detected *N* = 142	OR (95% CI)	*p* value	COVID − 19 Hospitalisation *N* = 54**	OR (95%CI)	*p* value	COVID-19 Death *N* = 10***	OR 95% CI	*p* value
Age^, *median (IQR)*	59 (20)	54 (20)		< 0.001	60 (18)		< 0.001	64.5 (14)		0.017
Male Sex (%)	172 (57)	95 (67)	1.5 (1.01–2.32)	0.046	38 (70)	1.3 (0.62–2.68)	0.49	7 (70)	1.2 (0.29–4.73)	1.0*
BMI^, *median (IQR)∼*	26.6 (8)	27.0 (8)		0.30	28.0 (7)		0.67	28.2 (11)		0.29
Place of birth				0.006			0.003			0.90
Australia and New Zealand	131 (43)	48 (34)			10 (19)			2 (20)		
Pacific Islander	16 (5)	17 (12)			13 24)			1 (10)		
Asia	67 (22)	39 (28)			14 (26)			4 (40)		
North/South America	13 (4)	3 (2)			2 (4)			2 (20)		
Middle East/Africa	33 (11)	24 (17)			10 (19)			0 (0)		
Europe	42 (14)	11 (8)			5 (9)			1 (10)		
Place of Birth										
- Pacific Islander	16 (5)	17 (12)	2.4 (1.12–4.97)	0.012	13 (24)	6.7 (2.04–21.70)	< 0.001	1 (10)	0.8 (0.10–6.79)	1.0*
Interpreter required	78 (26)	41 (29)	1.2 (0.75–1.82)	0.50	17 (32)	1.2 (0.58–2.58)	0.59	5 (50)	2.7 (0.73–9.76)	0.13
Smoking status ∼										
Never	183 (61)	94 (66)	0.9 (0.56-	0.46	32 (60)	1.6 (0.79-	0.18	2 (20)	8.3 (1.64-	0.006*
Former and Current	105 (35)	46 (33)	1.31)		21 (40)	3.34)		7 (78)	41.53)	
**CO-MORBIDITIES (%)**										
Hypertension	256 (85)	120 (85)	1.0 (0.56–1.70)	0.94	45 (83)	0.9 (0.34–2.19)	0.76	8 (80)	0.7 (0.14–3.61)	0.65*
Diabetes	149 (49)	72 (51)	1.1 (0.71–1.57)	0.79	34 (63)	2.2 (1.12–4.48)	0.022	8 (80)	4.3 (0.87–20.77)	0.097*
Cardiac Disease	79 (26)	42 (30)	1.2 (0.76–1.85)	0.45	21 (39)	2.0 (0.97–4.23)	0.057	4 (40)	1.6 (0.44–6.17)	0.48*
Respiratory Disease	67 (22)	22 (16)	0.6 (0.38–1.09)	0.10	11 (20)	1.8 (0.72–4.47)	0.21	5 (50)	6.8 (1.77–25.84)	0.002
Peripheral vascular disease	15 (5)	7 (5)	1.0 (0.40–2.50)	0.99	6 (11)	10.9 (1.27-93.00)	0.012*	3 (30)	13.7 (2.56–73.52)	0.008*
Neurological disease	78 (26)	28 (20)	0.7 (0.43–1.15)	0.16	12 (22)	1.3 (0.56–2.98)	0.56	3 (30)	1.8 (0.40–7.60)	0.41*
Haematological disease	46 (15)	19 (13)	0.9 (0.48–1.53)	0.61	6 (11)	0.7 (0.26–2.03)	0.53	2 (20)	1.7 (0.33–8.64)	0.62*
Liver disease	25 (8)	10 (7)	0.8 (0.39–1.80)	0.65	5 (9)	1.7 (0.47–6.15)	0.42	0	-	1.00*
Chronic Hepatitis B	11 (4)	7 (5)	1.4 (0.52–3.62)	0.52	4 (7)	2.3 (0.49–10.54)	0.43*	0	-	1.00*
Hepatitis C	2 (1)	3 (2)	3.2 (0.54–19.60)	0.33*	0	-	0.29*	0	-	1.00*
Autoimmune disease	33 (11)	12 (9)	0.8 (0.38–1.51)	0.42	4 (7)	0.8 (0.23–2.80)	1.0*	1 (10)	1.2 (0.14–10.56)	0.60*
Current Malignancy	27 (9)	6 (4)	0.4 (0.18–1.11)	0.087	0	-	0.083*	0		1.00*
Egfr start, *Mean (SD)* #	58 (21.4)	53 (22.6)		0.016	45.3 (19.7)		< 0.001	48 (22.5)		0.43
**RENAL FAILURE HISTORY**										
Dialysis modality ∼										
Peritoneal Dialysis	90 (30)	29 (21)		0.15	7 (13)		0.018	1 (10)		0.25
Haemodialysis	116 (39)	58 (41)			30 (57)			7 (70)		
Both	66 (22)	41 (29)			14 (26)			1 (10)		
Pre-emptive	25 (8)	13 (9)			2 (4)			1 (10)		
Dialysis Modality ∼										
HD	116 (38)	58 (41)	1.1 (0.74–1.66)	0.62	30 (56)	2.7 (1.33-	0.005	7 (70)	3.7 (0.92-	0.091*
Other	186 (62)	84 (59)			24 (44)	5.39)		3 (30)	14.99)	
Modality change	5 (2)	3 (2)	1.2 (0.30–5.44)	0.72*	2 (2)	0.8(0.07–9.17)	1.00*	0	-	1.0*
Primary Cause of Renal Failure										
Glomerulonephritis	152 (50)	70(49)		0.31	20 (37)		0.087	4 (40)		0.88
Diabetes	37 (12)	29 (20)			17 (32)			3 (30)		
ADPCKD	33 (11)	14 (10)			4 (7)			1(10)		
Obstructive Uropathy	26 (9)	10 (7)			4 (7)			1 (10)		
Hypertension	25 (8)	9 (6)			5 (9.3)			1 (10)		
Other	29 (10)	10 (7)			4 (7.4)			0 (0)		
**TRANSPLANT**										
Transplant Timing										
Other	277 (92)	129 (91)	1.1 (0.55–2.25)	0.76	52 (96)	0.3(0.06-	0.13*	9 (90)	1.1 (0.13-	1.0*
Pre Emptive	25 (8)	13 (9)			2 (4)	1.27)		1 (10)	9.53)	
Donor Type ∼										
Live	90 (30)	39 (27)	1.2 (0.74–1.81)	0.51	5 (9)	6.2 (2.24-	< 0.001	2 (20)	0.6 (0.32-	0.72*
Deceased	205 (68)	103 (73)			49 (91)	17.03)		8 (80)	7.68)	
Number of transplants										
1	290 (96)	139 (98)	1.9 (0.53–6.90)	0.41*	52 (96)	0.3 (0.03-	0.56*	10 (100)		1.0*
2 or 3	12 (4)	3 (2)			2 (4)	3.38)		0 (0)		
Mismatches∼										
0–1	20 (7)	8 (6)		0.40	2 (4)		0.71	0		0.53
2–3	65 (22)	29 (20)			11 (24)			3 (38)		
4–6	138 (46)	83 (58)			33 (72)			5 (63)		
Transplant vintage months, *median (IQR) ^*	75.5 (115)	54.5 (96)		0.003	55.5 (113)		0.73	81.5 (119)		0.52
**MEDICATIONS**										
Prednisolone	274 (91)	137 (97)	2.8 (1.06–7.41)	0.031	51 (94)	0.4 (0.06–2.45)	0.37	10 (100)	-	1.00*
Prednisolone (mg/day) *median (IQR)*	5.0 (5)	6.0 (5)		0.008	7.0 (5)		0.80	5 (3)		0.061
MMF	245 (81)	111 (78)	0.8 (0.51–1.36)	0.47	41 (76)	0.8 (0.36–1.83)	0.61	7 (70)	0.6 (0.15–2.59)	0.46*
MMF (mg/day) # *mean (SD)*	1166 (493)	1244 (499)		0.17	1094 (416)		0.015	1411 (342)		0.36
Tacrolimus	206 (68)	115 (81)	2.0 (1.22–3.22)	0.005	43 (80)	0.9 (0.37–2.04)	0.75	6 (60)	0.3 (0.08–1.21)	0.096*
Tac level (ng/ml) ^, *Median (IQR)*	6.1 (3)	6.4 (3)		0.33	7.1 (4)		0.31	7.9 (2)		0.16
Ciclosporin	35 (12)	8 (6)	0.5 (0.21–1.01)	0.048	4 (7)	1.7 (0.40–7.02)	0.48*	1 (10)	2.0 (0.22–17.94)	0.45*
CYA level (ng/ml)^ *median (IQR)*	85.0 (154)	263.0 (360)		0.023	314.0 (651)		0.86	-		1.0
Any CNI	241 (80)	123 (87)	1.7 (0.94–2.87)	0.081	47 (87)	1.1 (0.39–2.88)	0.91	7 (70)	0.3 (0.08–1.37)	0.13*
Everolimus	28 (9)	14 (10)	1.1 (0.55–2.10)	0.84	5 (9)	0.9 (0.28–2.83)	0.85	1 (10)	1.0 (0.12–8.68)	1.0*
Everolimus level (ng/ml (# *mean (SD)*	5.2 (2.0)	5.5 (1.3)		0.67	5.5 (1.5)		0.98	4.0(-)		0.25
Sirolimus	19 (6)	3 (2)	0.3 (0.09–1.11)	0.058	1 (2.0)	0.8 (0.07–9.17)	1.00*	0	-	1.0*
Sirolimus level (ng/ml) # *mean (SD)*	6.1 (1.4)	4.5 (2.2)		0.12	-		0.16	-		-
Any mTOR	47 (16)	17 (12)	0.7 (0.41–1.34)	0.32	6 (11)	0.9 (0.30–2.52)	0.81	1 (10)	0.8 (0.10–6.79)	1.0*
Azathioprine	8 (6)	31 (10)	0.5 (0.23–1.17)	0.11	3 (6)	1.0 (0.22–4.26)	1.0*	1 (10)	1.9 (0.22–17.94)	0.45*
Azathioprine dose (mg/day) # *mean (SD)*	85 (36.2)	75 (42.3)		0.49	75 (43.3)		1.0	-		0.23
ATG	29 (10)	17 (12)	1.2 (0.68–2.42)	0.45	5 (9)	0.6 (0.21–1.95)	0.44	0 (0)		0.61*
Time to ATG (years) ^, *median(IQR)*	3.0 (7)	2.0 (5)		0.14	6.0 (7)		0.027	0 (0)		-
Use of any Blood Thinner	98 (33)	37 (26)	0.7 (0.47–1.15)	0.17	20 (37)	2.5 (1.14–5.28)	0.020	4 (40)	2.0 (0.53–7.53)	0.29*
**VACCINATION HISTORY**										
Vaccination∼										
0–2 Doses	53 (18)	43 (30)	0.5 (0.31–0.79)	0.003	19 (35.2)	0.7 (0.33-	0.32	6 (60.0)	0.3	0.067*
3–4 Doses	245 (81)	99 (70)			35 (64.8)	1.43)		4 (40.0)	(0.070–0.97)	
Seroconversion∼∼	24 (40.0)	3 (13)	0.2 (0.06–0.84)	0.020*	0	-	0.53	-		-
**COVID-19 THERAPIES**										
Sotrovimab	-	62 (44)	-	-	19 (35.2)	0.6 (0.28–1.14)	0.11	0		0.005*
Time to sotrovimab (days) *median (IQR) ^*	-	1 (3)	-	-	1 (3)		0.88			
Molpurinovir	-	11 (8)	-	-	2 (4)	0.3 (0.07–1.63)	0.21*	0		1.00*

∼ Missing: BMI = 16, Smoking = 16, Dialysis modality = 6, Donor type = 7, Mismatches 101, Total vaccination = 4,

∼∼Seroconversion subgroup *n* = 83

^ Mann Whitney U, # independent sample t-test

*Fischer’s exact test

** as compared to those with COVID-19 who were not hospitalised *** as compared to those with COVID-19 who survived

IQR– interquartile range, SD- Standard Deviation, CI- confidence interval. OR– odd ratio

Totals may not be 100% due to rounding or missing data

### COVID-19 outcomes

COVID-19 was reported in 142 (32%) patients, and of these 54 (38%) required admission for COVID-19 with 10 (7%) deaths due to COVID-19. 17 (4%) patients died from any cause during the study period, with COVID-19 accounting for 59% of all deaths.

### COVID-19 diagnosis

Univariate factors associated with acquiring COVID-19 are shown in Table 2. On multivariable analysis, an increased risk of acquiring COVID-19 was associated with male sex (aOR 1.7, 1.093–2.701, *p* = 0.019), younger age (aOR, 0.98, 0.964–0.994, *p* = 0.006) and lower eGFR (aOR 0.99, 0.978–0.998, *p* = 0.020), after adjusting for significant univariate associations, body mass index (BMI) and diabetes. (Table 3). Receipt of 3 or more doses of vaccine was protective (aOR 0.48, 95% CI 0.287–0.796, *p* = 0.005).

**Table 3:** Multivariable Binary Logistic Regression Models

Regression Models	aOR	95% CI	*P*
**COVID-19 Diagnosis**			
Younger Age	0.98	0.964–0.994	0.006
Male Sex	1.7	1.093–2.701	0.019
Lower eGFR as study start	0.99	0.978–0.998	0.020
Vaccination 3 + doses	0.48	0.287–0.796	0.005
**COVID-19 Hospitalisation**			
Older Age	1.0	1.007–1.092	0.021
eGFR at study start	0.96	0.934–0.982	< 0.001
Deceased donor graft	4.1	1.128–14.747	0.032
**COVID-19 Mortality**			
Older Age	1.1	1.004–1.192	0.040
Respiratory disease	14.2	1.825–110.930	0.011
Current and Former Smoker	8.2	1.020-65.649	0.048
Vaccination 3 + doses	0.6	0.007–0.523	0.011
**Seroconversion Subgroup**			
**COVID-19 Diagnosis**			
Asian place of birth	9.0	1.803–44.888	0.007
Higher Dose of prednisolone	1.5	1.125–1.949	0.005
Seroconversion	0.1	0.025–0.627	0.011

In Model:

Diagnosis: Age, BMI, Gender, Diabetes, Pacific Place of birth, eGFR at start, Transplant vintage (months), dose of prednisolone, Tacrolimus present, Cyclosporin present, Vaccination (3 + doses)

Hospitalisation: Age, BMI, Gender, Diabetes, Pacific place of birth, eGFR at start, Haemodialysis (vs. other prior modality), Deceased donor graft (vs. other), Dose of Mycophenolate, Use of Blood thinner, Vaccination (3 + doses)

*Note. *Time to ATG and Peripheral vascular disease variables excluded due to model limitations of total variable number, model instability and wide confidence intervals

Mortality: Age, BMI, Gender, Diabetes, Respiratory disease, Peripheral vascular disease, Smoking status, Vaccination (3 + doses)

In Model (Seroconversion Subgroup):

Age, Place of Birth, Dose of Prednisolone, Diabetes, mTOR present, CNI present, Transplant vintage (months), Vaccination of 3 + doses, Seroconversion status

### COVID-19 mortality

Deaths from COVID-19 occurred throughout the study period, with 3 deaths in September 2021, 1 death in January 2022, 4 deaths in February 2022 and 1 death in both April and May 2022. Univariate analyses are shown in Table 2. On multivariate analysis, increased mortality due to COVID-19 was associated with older age (aOR1.1, 95%CI 1.004–1.192, *p* = 0.04), respiratory disease (aOR 14.2, 95%CI 1.825–110.930, *p* = 0.011) and current or past smoking exposure (aOR 8.2, 95% CI 1.020-65.649, *p* = 0.048) after adjusting for significant univariate associations, sex, BMI, diabetes, and vaccination (3 + doses). Vaccination of 3 or more doses was protective (aOR 0.6, 95% CI 0.007–0.523, *p* = 0.011) (Table 3).

### COVID-19 hospitalisation

Of those with reported COVID-19, 62 (44%) received sotrovimab and 11 (8%) received molnupiravir (Suppl Table 6.). 54 (38%) patients required hospitalisation for COVID-19, and 16 (11%) required intensive care unit (ICU) care. 33 (23%) patients required oxygen therapy. The maximum level of oxygen required was: low flow nasal prong oxygen in 13 (9%), high flow nasal prong oxygen in 4 (3%), non-invasive ventilation in 8 (6%) and invasive ventilation in 8 (6%) patients. Sotrovimab and molnupiravir were given in the community. When provided, neither were found to be protective for hospital admission (*p* = 0.11, *p* = 021 respectively). Among hospitalised patients, those who received sotrovimab had evidence of protection for ICU admission (OR 0.2, 95%CI 0.035–0.886, *p* = 0.030). Median length of hospital stay was 8 days (IQR ± 13). There was an association between prior sotrovimab use and shorter length of stay (5 vs. 10 days, *p* = 0.027). Vaccination with 3 doses did not impact hospital admission (*p* = 0.32), ICU admission (*p* = 0.14) or length of stay (0.54).

Immunosuppression alteration occurred frequently in hospitalised patients (85%), as compared to those who were not hospitalised (10%). Hospitalisation with COVID-19 increased the odds of a reduction of immunosuppression (OR 50.5, 95% CI 18.211-139.883, *p* < 0.001), however it was not significant for those who required an ICU admission among hospitalised patients (*p* = 0.41) or mortality (*p* = 0.64). Univariate factors associated with hospitalisation for COVID-19 are shown in Table 2.

On multivariable analysis, increased hospitalisation was associated with older age (aOR 1.0, 95% CI 1.007–1.0092, *p* = 0.021), lower eGFR (aOR 0.96, 95% CI 0.994 − 0.982, *p* < 0.001) and receipt of a deceased donor graft (aOR 4.1, 95% CI 1.128–14.747, *p* = 0.032), after adjusting for significant univariable associations, sex, BMI and vaccination (3 doses) (Table 3). Vaccination was not protective.

### Seroconversion

84 patients underwent serological testing, including: 71 patients who had a single test, 12 who had 2 serial tests and 1 patient who had 3 serial tests. All but one patient, had a non-reactive Eleycs assay, indicating no prior exposure to COVID-19. The single patient with a reactive Eleycs assay was not known to have had prior COVID-19, however, was transplanted overseas with limited details prior to returning to Australia before the study period. This patient had no further serological evaluation and was excluded from the seroconversion analysis, resulting in 83 patients providing 97 serological tests assessed for vaccine-induced seroconversion.

All but 2/97 tests were collected prior to documented COVID-19. These two patients participated in serial testing. Prior to known COVID-19 they were Elecsys assay and QuantiVac ELISA negative. Post COVID-19 they remained Elecsys assay negative, however seroconverted on the QuantiVac ELISA. During this interval they received additional vaccinations, incrementing from 2 to 3 doses. As it is not possible to determine if these patients seroconverted due to wild type COVID-19 infection or vaccination, the serial samples prior to known COVID-19 were analysed. Of the remaining 95 tests, 5 were excluded based on QuantiVac ELISA results: 1 patient who did not have a QuanitVac ELISA processed on initial collection 1, but undertook repeat testing which was utilised, 3 patients with serial reactive tests performed after 2 and 3 doses of vaccine with no status change, therefore sampling after the 2nd dose was included, and 1 patient with 2 serial reactive tests, both after the 4th dose of vaccine and the earlier sample was included.

This resulted in 90 analysed samples: 1, 64, 21 and 4 samples after 1,2,3 and 4 doses of vaccine respectively (Suppl Table 3). Seroconversion rates after 1, 2, 3 and 4 doses were: 0, 22%, 48%, and 75% respectively (Suppl Table 4). Overall seroconversion rate at study end was 33% (27/83).

Univariate factors associated with COVID-19 diagnosis in this subgroup are shown in Supplementary Table 5. On multivariable analysis, after adjusting for univariate associations, in addition to age and diabetes, factors associated with an increased rate of acquiring COVID-19 included Asian place of birth (aOR 9.0, 95% CI 1.803–44.888, *p* = 0.007) and higher dose of prednisolone (aOR 1.5, 95% CI 1.125–1.949, *p* = 0.005). Seroconversion was protective (aOR 0.1, 95% CI 0.025–0.627, *p* = 0.011), independent of vaccination of 3 + doses (*p* = 0.108) (Table 3).

The number of hospitalised patients in this subgroup was small (*n* = 6). No hospitalised patients demonstrated evidence of seroconversion, however this did not reach statistical significance (*p* = 0.539). No patient who died underwent serology assessment.

## Discussion

In this large, observational study of KTRs in Australia, during a period following community stay-at-home orders and two strain outbreaks, COVID-19 resulted in significant morbidity and mortality throughout the 2021–2022 pandemic period. Over 30% of the cohort developed breakthrough COVID-19, despite 78% receiving 3 or more doses of vaccine. Early monoclonal or antiviral treatment was provided to 51% of positive patients, however 38% of patients still required hospitalisation, with death occurring in 7% [15, 16]. Overall seroconversion rates were low, with 3 doses of vaccine achieving a seroconversion rate of 48%.

Several risk factors for mortality amongst KTRs have been suggested, including older age, sex, cardiometabolic or respiratory co-morbidities and obesity [1, 3–7, 11, 23].. This data supports older age, respiratory disease and smoking exposure may be independent factors for mortality for COVID-19 in KTRs. On systematic review and registry data analysis, no single co-morbidity had consistently been identified as a risk factor, other than age [3, 11].

It has been suggested certain immunosuppression regimens are associated with increased COVID-19 mortality [1, 6, 7]. We did not find any effect of individual immunosuppressive agent on mortality, however there were high rates of baseline steroid (93%) and anti-metabolite (89%) use. A higher dose of prednisolone was associated with increased risk of acquiring COVID-19 in the serology subset.

Prior recommendations suggested temporarily altering immunosuppressive regimens during COVID-19 infections, and we noted high rates of alteration on hospitalisation in line with this trend [29]. There was no association with ICU admission or mortality among this group, and of patients who were not hospitalised, the majority did not have drug alteration (90%). Drug alteration, therefore, is likely reflective of a response to the severity of COVID-19. Current advice, with new strain evolution, suggests immunosuppression alteration is not required, particularly in the asymptomatic or those with a mild illness, with our data supportive of this [30]. Systematic review has not supported an association between immunosuppression and mortality, and there is limited comparative data to guide reduction of immunosuppression therefore decisions should be based on individualized assessment and the risk of rejection [11, 23].

This data covered a period until May 2022, during a predominant Omicron outbreak from January 2022, whereby most patients had received 3 or more vaccine doses [15, 16].. The Omicron era heralded decreased virulence, however the neutralising capability after 3 doses of vaccine was suggested to be diminished [12]. In this data, vaccination of 3 or more doses was protective for death and acquiring COVID-19, with no effect on hospitalisation, ICU admission or length of stay. In the serology subset, seroconversion, independent of dose of vaccination, was protective for acquiring COVID-19. Mortality rates during periods of Omicron predominance among solid organ transplant recipients have been reported to be 3 − 4%, however hospitalisation rates have remained 24–32%, with ICU admission rates of 28–36% [24, 31]. Ongoing hospitalisation rates remain a concern for KTRs and further data regarding vaccine schedule optimisation and seroconversion assessment, independent of vaccination dose number, is needed.

This data demonstrated protection against death, reduced rates of ICU admission and length of stay with the use of sotrovimab, with no protective effects of molnupiravir. Our study reflects a period where sotrovimab was the primary agent of choice in early COVID-19 disease (approved August 2021) as opposed to molnupirovir (approved January 2022), likely influencing our results [27, 28].

Current Australian recommendations do not recommend either sotrovimab or molnupirovir. Tixagevimab plus cilgavimab (Evusheld) has also lost its recommendation. Nirmatrelvir plus ritonavir (Paxlovid) retains its conditional recommendation, however, its use in KTRs is challenging due to effects on calcineurin inhibitor levels [25]. Remdesivir remains recommended only for patients requiring oxygen due to symptomatic COVID-19. There remains a paucity of agents effective at treating early COVID-19 in renal transplant recipients.

This data supports concern surrounding ongoing mortality and hospitalisation risk for KTRs, in the context of low seroconversion rates despite increasing vaccination dose schedule. This reiterates vaccination of at least 3 doses, and potentially evidence of seroconversion, is protective, however, in the absence of effective early treatments, encouragement of protective behaviours, such as social distancing, mask compliance and hand hygiene should continue.

This study is limited as a single centre and results are not generalisable. As with all observational data our analyses are limited to associations. Those undertaking the serology assessment were a self-selected population, which is likely to result in unmeasured patient bias, especially with regards to protective behaviours. They were highly vaccinated, with more than 90% receiving 3 or more doses. In addition, our data spanned two predominant strain periods, Delta and Omicron, and we were not able to specify strains in individual patients. While it is likely we captured most noted infections due to mandatory government reporting, cases could have been omitted if patients did not note an infection, obtain testing, or report a positive test, resulting in potential underdiagnosis of mild and asymptomatic cases. In addition, seroconversion does not always reflect in-vivo activity of antibodies and we did not assess the effect of waning immunity over time.

### Electronic supplementary material

Below is the link to the electronic supplementary material.

Supplementary Material 1

## Data Availability

The datasets used and/or analysed during the current study are available from the corresponding author on reasonable request.
