# Supplementary material for: A prospective observational cohort study of covid-19 epidemiology and vaccine seroconversion in South Western Sydney, Australia, during the 2021–2022 pandemic period
Source: BMC Nephrol. 2024 Apr 12;25:131. doi: 10.1186/s12882-024-03560-8 (PMC11015631; doi:10.1186/s12882-024-03560-8)
Supplement: Supplementary file 1 — Supplementary Material 1 [file 12882_2024_3560_MOESM1_ESM.docx]

**SUPPLEMENTARY INFORMATION**

**Supplementary Table 1**

| **Elecsys Anti-Sars-CoV-2** | **QuantiVac ELISA** | **Infection status** | **Vaccination Status** | **Result** |
| --- | --- | --- | --- | --- |
| Negative | Negative | - | - | No infection or evidence of seroconversion |
| Positive | Negative | - | - | Wild type infection |
| Negative | Positive | No known infection | Known vaccination | Vaccine-induced seroconversion |
| Negative | Positive | Known infection | No vaccination | Wild type infection |
| Negative | Positive | Known infection | Known vaccination | Either wild-type infection or vaccine-induced seroconversion |
| Positive | Positive | - | - | Wild Type infection |

**Table 1. S**erology results and clinical decision making based on prior infection and vaccination status

**Supplementary Table 2** - **Breakdown of Vaccine types for dose 1, 2 3 4**

| **Brand** | **Dose 1 (%)**  **N= 423** | **Dose 2 (%)**  **N= 419** | **Dose 3 (%)**  **N= 344** | **Dose 4 (%)**  **N= 105** | **Total (%)**  **N= 1291** |
| --- | --- | --- | --- | --- | --- |
| **Astrazeneca** | 165 (39.0) | 161 (38.4) | 6 (1.7) | 0 | **332 (26)** |
| **Pfizer** | 253 (59.8) | 253 (60.4) | 309 (90.0) | 93 (88.6) | **908 (70)** |
| **Moderna** | 4 (1.0) | 4 (1.0) | 26 (7.5) | 10 (9.5) | **44 (3)** |
| **Unknown** | 1 (0.2) | 1 (0.2) | 3 (0.8) | 2 (1.9) | **7 (1)** |

**SEROCONVERSION Testing**

**SEROCONVERSION SAMPLES for assessment – N = 98**

**Excluded if:**

1. Serial sample and evidence of prior seroconversion and no status change e.g

0>1>1: count after Vax 2

0>0>1: count after Vax 3

1. Interval COVID-19 + increasing Vax dose and unable to differentiate effects in later sample
2. Evidence of prior wild-type COVID-19

**Supplementary Table 3 – Summary of selection of serology samples for analysis**

Excluded: Elecsys positive. Presumed earlier wild type infection (#253)

| **Vaccine #** | **RAW SAMPLES**  **N = 97** | **SAMPLES USED**  **N = 90** | **REASON** |
| --- | --- | --- | --- |
| 1 | 1 | 1 |  |
| 2 | 65 | 64 | No quantiVac processed (#453) on Collection 1. Patient underwent second collection and later sample utilised. |
| 3 | 26 | 21 | 3 serial samples with evidence of seroconversion after 2 doses of Vaccine and no status change (#52,56,440)  2 serial samples with seroconversion but also interval COVID-19, keep pre-Covid-19 sample (#327, #223) |
| 4 | 5 | 4 | 1 serial samples, both after 4 doses, from same pt with no change(#28) |

**Supplementary Table 4. Seroconversion results by Vaccine dose**

|  | **Vaccine 1**  **( N=1)** | **Vaccine 2**  **(n=64)** | **Vaccine 3**  **(n=21)** | **Vaccine 4**  **(n=4)** | **Total** |
| --- | --- | --- | --- | --- | --- |
| No | 1 (100) | 50 (78) | 11 (52) | 1 (25) | - |
| Yes + Borderline | 0 | 13 +1 (22) | 9 +1 (48) | 3 (75) | 27 |

**Seroconversion for total cohort of patient (n= 83)**

27/83 patients (when counting 1 sample per patient) = 33%

**Supplementary Table 5 – Univariate factors association with COVID-19 diagnosis and hospitalisation: Serology Subgroup**

| **Variable** | **No COVID-19 Detected**  **N=60** | **COVID -19 Detected**  **N= 23** | **OR (95% CI)** | **p-value** | **COVID-19 Hospitalisation**  **N = 6** | **OR (95% CI)**** | **p-value** |
| --- | --- | --- | --- | --- | --- | --- | --- |
| **CHARACTERISTICS** |  |  |  |  |  |  |  |
| Age^ , *median (IQR)* | 60 (17) | 54 (16) | - | 0.051 | 56 (21) | - | 0.32 |
| Male Sex (%) | 33 (55) | 11 (48) | 0.8 (0.29-1.97) | 0.56 | 5 (83) | 9.2 (0.86-97.69) | 0.07* |
| BMI^, *median (IQR)* | 26.3 (6) | 25.0 (8) | - | 0.66 | 27.7 (17) | - | 0.526 |
| Place of birth  Australia and New Zealand  Pacific Islander  Asia  North/South America  Middle East/Africa  Europe | 32 (53)  3 (5)  11 (18)  2 (3)  5 (8)  7 (12) | 11 (48)  0  9 (39)  0  1 (4)  2 (9) |  | 0.36 | 2 (33)  -  3(50)  -  1 (17)  0 (0) |  | 0.25 |
| Place of Birth   - Asia vs Other | 11 (18) | 9 (39) | 2.9 (0.99-8.29) | 0.047 | 3 (50) | 1.8 (0.28-12.07) | 0.64* |
| Interpreter required | 11 (18) | 3 (13) | 0.7(0.17-2.65) | 0.75* | 1 (17) | 1.5 (0.11-20.30) | 1.00* |
| Smoking status~  Never  Former and Current | 36 (62)  22 (38) | 14 (61)  9 (39) | 1.1 (0.39-2.84) | 0.92 | 2 (33)  4 (67) | 4.8 (0.66-35.20) | 0.16* |
| **CO-MORBIDITIES** |  |  |  |  |  |  |  |
| Hypertension (%) | 57 (95) | 20 (87) | 0.4 (0.07-1.88) | 0.34* | 6 (100) | - | 0.54* |
| Diabetes | 23 (38) | 9 (39) | 1.0 (0.39-2.77) | 0.95 | 4 (67) | 4.8 (0.66-35.20) | 0.16* |
| Diabetes  Type 1  Type 2 | 7 (30)  16 (70) | 1 (11)  8 (89) | 6.5 (0.37-33.56) | 0.39* | 1 (25)  3 (75) | - | 0.44 |
| Cardiac Disease | 13 (22) | 7 (30) | 1.6 (0.54-4.66) | 0.40 | 2 (33) | 1.2 (0.16-8.80) | 1.00* |
| Respiratory Disease | 15 (25) | 3 (13) | 0.5 (0.12-1.73) | 0.37* | 1 (17) | 1.5 (0.11-20.30) | 1.00* |
| Peripheral vascular disease | 6 (10) | 2 (9) | 0.9 (0.16-4.59) | 1.00* | 2 (33) | - | 0.06* |
| Neurological disease | 14 (23) | 6 (26) | 1.2 (0.38-3.51) | 0.79 | 1 (17) | 0.5 (0.04-5.22) | 1.00* |
| Haematological disease | 9 (15) | 5 (22) | 1.6 (0.47-5.32) | 0.46 | 0 (0) | - | 0.27* |
| Liver disease | 3 (5) | 1 (4) | 0.9 (0.09-8.75) | 1.00* | 0 (0) | - | 1.00* |
| Hepatitis B | - | - | - | - | - | - | - |
| Hepatitis C | - | - | - | - | - | - | - |
| Autoimmune disease | 11 (18) | 1 (4) | 0.2 (0.03-1.67) | 0.17* | 1 (17) | - | 0.26* |
| Current Malignancy | 6 (10) | 2 (9) | 0.9 (0.16-4.59) | 1.00* | 0 (0) | - | 1.00* |
|  |  |  |  |  |  |  |  |
| Egfr start, *Mean (SD)* # | 58 (21) | 55 (20) | - | 0.55 | 52 (10) | - | 0.61 |
| **RENAL FAILURE HISTORY** |  |  |  |  |  |  |  |
| Dialysis modality  Peritoneal Dialysis  Haemodialysis  Both  Pre-emptive | 23 (38)  19 (32)  13 (22)  5 (8) | 4 (17)  9 (39)  8 (35)  2 (9) |  | 0.30 | 0 (0)  4 (67)  1 (17)  1 (17) |  | 0.23 |
| Modality change | 1 (2) | 0 | - | 1.00* | - | - | - |
| Primary Cause of Renal Failure  Glomerulonephritis  Diabetes  ADPCKD  Obstructive Uropathy  Hypertension  Other | 32 (53)  8 (13)  7 (12)  5 (8)  5 (8)  3 (5) | 18 (78)  2 (9)  2 (9)  0  0  1 (4) |  | 0.31 | 4 (67)  2 (33)  0 (0)  -  -  (0) |  | 0.08 |
| **TRANSPLANT** |  |  |  |  |  |  |  |
| Transplant Timing  Other  Pre-Emptive | 55 (92)  5 (8) | 21 (91)  2 (9) | 1.0 (0.19-5.82) | 1.00* | 5 (83)  1 (17) | 3.2 (0.17-61.012) | 0.46* |
| Donor Type  Live  Deceased | 19 (32)  41 (68) | 5 (22)  18 (78) | 1.7 (0.54-5.17) | 0.37 | 1 (17)  5 (83) | 1.5 (0.14-17.33) | 1.00* |
| Number of transplant   - 2/3 - 1 | 59 (98)  1 (2) | 21 (91)  2 (9) | 5.6 (0.02-2.07) | 0.18* | 1 (17)  5 (83) | 0.3 (0.02-5.96) | 0.46* |
| Mismatches group  0-1  2-3  4-6 | 3 (7)  12 (27)  30 (67) | 2 (10)  3 (14)  16 (76) | - | 0.52 | 2 (33)  0 (0)  4 (67) | - | 0.04 |
| Transplant vintage months, *median (IQR) ^* | 62.0 (103) | 33.0 (54) | - | 0.02 | 17.5 (68) | - | 0.56 |
| **MEDICATIONS** |  |  |  |  |  |  |  |
| Prednisolone | 56 (93) | 22 (96) | 1.6 (0.17-14.85) | 1.00* | 6 (100) | - | 1.00* |
| Dose Pred^, *median (IQR)* | 5.0 (5) | 9.0 (5) |  | 0.005 | 9.0 (7) |  | 0.49 |
| MMF | 50 (83) | 17 (73.9) | 0.6 (0.18-1.79) | 0.33 | 4 (66.7) | 0.6 (0.08-4.70) | 0.63* |
| Dose MMF# , *mean (SD)* | 1188.2 (425) | 1223 (522) |  | 0.78 | 1110 (464) |  | 0.64 |
| Tacrolimus | 42 (70) | 20 (87) | 2.9 (0.75-10.84) | 0.16* | 5 (83) | 0.7 (0.05-9.02) | 1.00* |
| Tac level^, *median(IQR)* | 6.4 (4) | 7.3 (3) |  | 0.22 | 8.7 (8) |  | 0.45 |
| Ciclosporin | 3 (5.0) | 2 (9) | 1.8 (0.28-11.60) | 0.61* | 1 (17) | 3.2 (0.17-61.02) | 0.46* |
| CYA level^ *median (IQR)* | 52.0 (-) | - |  | 1.00 | - |  | - |
| CNI vs other | 45 (75) | 22 (96) | 7.3 (0.91-59.14) | 0.03* | 6 (100) | - | 1.00* |
| Everolimus | 8 (13) | 1 (4) | 0.3 (0.04-2.51) | 0.43* | 0 (0) | - | 1.00* |
| Everolimus level#, *mean (SD)* | 4.2 (2.3) | 5.0 (-) |  | 0.76 | - |  | - |
| Sirolimus | 8 (13) | 0 (0.0) | - | 0.01* | - | - | - |
| Sirolimus level#, *mean (SD)* | 6.7 (1.1) | - |  | - | - |  | - |
| mTOR Any | 16 (27) | 1 (4) | 0.1 (0.02-1.01) | 0.032* | 0 (0) | - | 1.00* |
| Azathioprine | 4 (7) | 0 (0) | - | 0.57* | - | - | - |
| Azathioprine dose#, *mean (SD)* | 2.2 (67) | - |  | - | - |  | - |
| ATG | 4 (7) | 5 (22) | 3.9 (0.94-16.06) | 0.11* | 1 (17) | 0.7 (0.06-7.32) | 1.00* |
| Time to ATG(years), *median(IQR)^* | 1.0 (1) | 2.0 (7) |  | 0.41 | - |  | 0.80 |
|  |  |  |  |  |  |  |  |
| Use of any Blood Thinner | 15 (25) | 5 (22) | 0.8 (0.26-2.63) | 0.76 | 2 (33) | 2.3 (0.28-19.17) | 0.58* |
|  |  |  |  |  |  |  |  |
| **VACCINATION** |  |  |  |  |  |  |  |
| Vaccination  0-2 Doses  3-4 Doses | 4 (7)  56 (93) | 2 (9)  21 (91) | 0.8 (0.13-4.40) | 0.67* | 0 (0)  6 (100) | - | 1.0* |
| Vaccination  0-3  4+ | 29 (48)  31 (52) | 18 (78)  5 (22) | 0.3 (0.09-0.79) | 0.01 | 3 (50)  3 (50) | 7.5 (0.85-66.13) | 0.09* |
| Seroconversion | 24 (40) | 3 (13) | 0.2 (0.06-0.84) | 0.02* | 0 (0) | - | 0.54* |
| **COVID-19 TREATMENT** |  |  |  |  |  |  |  |
| Sotrovimab | - | - | - | - | 2 (33) | 0.4 (0.05-2.47) | 0.37* |
| Time to sotrovimab (days) *median (SD) ^* | - | - | - | - | 1.5 (1) |  | 0.91 |
| Molpurinovir | - | - | - | - | 1 (17) | 1.5 (0.11-20.30) | 1.00* |

Totals may not reach 100% due to rounding or missing data

~ Missing: Smoking = 2

^ Mann Whitney U, # independent sample t-test.

*Fischers exact test

** as compared to those with COVID-19 who were not hospitalised

IQR – interquartile range. CI- confidence interval. OR – odd ratio

**Supplementary Table 6. Treatments and level of oxygen support for COVID-19**

| **Variable** | **N = 142 (%)** |
| --- | --- |
| Sotrovimab | 62 (44) |
| Molpurinovir | 11 (8) |
| COVID Admission | 54 (38) |
| ICU Admission | 16 (11) |
| Ventilatory Support  Nasal prong oxygen  High Flow Nasal Prong  Non-Invasive Ventilation  Intubation | 33 (23)  13 (9)  4 (3)  8 (6)  8 (6) |
| Dexamethasone | 31 (22) |
| Baricitinib | 8 (6) |
| Tocilizumab | 5 (4) |
| Remdesivir | 6 (4) |
| Sarilumab | 2 (1) |
| Evidence of reduced immunosuppression | 55 (39) |
